# Controlled delivery of β-globin-targeting TALENs and CRISPR/Cas9 into mammalian cells for genome editing using microinjection

**DOI:** 10.1038/srep16031

**Published:** 2015-11-12

**Authors:** Renee N. Cottle, Ciaran M. Lee, David Archer, Gang Bao

**Affiliations:** 1 Georgia Institute of Technology and Emory University, Department of Biomedical Engineering, Atlanta, GA 30332, USA; 2 Rice University, Department of Bioengineering, Houston, TX 77030, USA; 3 Emory University School of Medicine, Department of Pediatrics, Atlanta, GA 30322, USA

## Abstract

Tal-effector nucleases (TALEN) and clustered regularly interspaced short palindromic repeats (CRISPR) with CRISPR-associated (Cas) proteins are genome editing tools with unprecedented potential. However, the ability to deliver optimal amounts of these nucleases into mammalian cells with minimal toxicity poses a major challenge. Common delivery approaches are transfection- and viral-based methods; each associated with significant drawbacks. An alternative method for directly delivering genome-editing reagents into single living cells with high efficiency and controlled volume is microinjection. Here, we characterize a glass microcapillary-based injection system and demonstrate controlled co-injection of TALENs or CRISPR/Cas9 together with donor template into single K562 cells for targeting the human β-globin gene. We quantified nuclease induced insertions and deletions (indels) and found that, with β-globin-targeting TALENs, similar levels of on- and off-target activity in cells could be achieved by microinjection compared with nucleofection. Furthermore, we observed 11% and 2% homology directed repair in single K562 cells co-injected with a donor template along with CRISPR/Cas9 and TALENs respectively. These results demonstrate that a high level of targeted gene modification can be achieved in human cells using glass-needle microinjection of genome editing reagents.

Site-specific modification of endogenous genomic loci mediated by engineered nucleases has unprecedented potential for a wide array of applications, such as engineering model organisms^1^,^2^,^3^,^4^ and developing new therapeutic strategies^5^,^6^ Examples of site-specific nuclease platforms include zinc-finger nucleases (ZFNs), Tal-effector nucleases (TALENs) and clustered regularly interspaced short palindromic repeats (CRISPR) and CRISPR-associated (Cas) proteins. DNA double stranded breaks induced by engineered nucleases can be repaired by the error-prone non-homologous end joining (NHEJ) or the high fidelity homology directed repair (HDR) pathways, leading to genome alterations, such as gene knockout or reconstitution at a desired target site^7^. HDR guided by exogenous donor template DNA having homologous sequences on both sides of the break site can be exploited for gene correction of mutations causing diseases, such as sickle cell anemia^6^. The potential benefits of nuclease-mediated HDR are targeted gene correction instead of uncontrollable random gene integration, and enhanced levels of gene correction compared to delivering homologous donor template DNA alone into cells. Recently, modification of the human β-globin (*HBB*) locus was achieved using ZFNs^8^, TALENs^9^,^10^ and the CRISPR/Cas9 system^11^, demonstrating the potential for a nuclease-based gene correction approach for treating sickle cell anemia.

One major challenge for advancing nuclease-based therapeutic strategies is to deliver optimal levels of nucleases and donor DNA into clinically relevant cell types^12^. Specifically, if the amount of nuclease-encoding plasmid and donor DNA is too low, the HDR rate would be insufficient to have a reasonable level of gene correction. On the other hand, if the amount of nucleases in cells is too high, a large amount of cell death could occur due to cytotoxicity^13^,^14^,^15^. Thus, it is critical to optimize the delivery protocol so that both a high level of nuclease activity and low level of cytotoxicity could be achieved. Although viral-based methods have been used for the delivery of gene targeting reagents into cell lines^16^ and stem cells^17^,^18^, there are many concerns, including random vector insertion, immunogenicity, integrity of packaged vectors, and effects of extensive stem cell culturing^12^,^16^,^19^. The most deleterious safety issue associated with viral-mediated delivery is potential activation of proto-oncogenes leading to tumors as a result of random vector insertion^19^. Transfection-based methods, such as nucleofection, have been used as a nuclease delivery method into cells^5^,^18^, but cell loss due to cytotoxicity^18^ remains an issue. Furthermore, with transfection it is difficult to control the amount of nucleases and donor template delivered into single cells.

As an alternative, microinjection can be used for the direct delivery of nucleases and donor template into cells by penetrating the cell membrane using glass microcapillaries with fine tips. Glass microcapillary-mediated microinjection has been shown to deliver recombinant proteins, peptides, and DNA into a wide variety of human primary cell cultures and cell lines, including CD34^+^ cells^20^,^21^, neurons^22^,^23^,^24^,^25^,^26^,^27^,^28^, kidney 293 cells^29^, HeLa cells^30^, and fibroblasts^31^. There have been multiple studies demonstrating successful microinjection-based delivery of nucleases into embryos for the direct production of animal models with targeted mutations^32^,^33^,^34^. Although it is a low throughput technique, microinjection allows for the precise control of the amount delivered into single cells, and can achieve high (~100%) delivery efficiency^35^. For cells that are difficult to transfect, microinjection is an attractive alternative and is potentially less toxic and stressful to cells^35^.

Here we demonstrate microinjection-based delivery of nucleases into human K562 cells with resulting gene modifications. This study is the first, to our knowledge, to apply microinjection for delivering genome editing reagents into cultured cells. We characterized a glass microcapillary injection system and quantified the effects of the microinjection method on cell viability and proliferation. Microinjection of plasmids encoding an *HBB*-targeting TALEN pair and CRISPR/Cas9 system respectively into single K562 cells resulted in moderate to high levels of cleavage activities as quantified by the T7 endonuclease I (T7E1) mutation detection assay and Sanger sequencing. We also quantified HDR in single K562 cells co-injected with *HBB*-targeting TALENs or CRISPR/Cas9 along with a GFP donor template, and determined the off-target cleavage in the δ-globin (*HBD*) and glutamate receptor (*GRIN3A*) genes respectively. Our results suggest that microinjection of engineered nucleases, such as TALENs and CRISPR/Cas9 systems, can lead to high levels of gene editing. It is expected that the successful demonstration of nuclease-mediated gene editing in K562 cells using glass-needle microinjection may facilitate the development of high-throughput microinjection systems for genome editing in living cells.

## Results

### Microinjection on retronectin-coated polystyrene dishes

**Figure 1:**
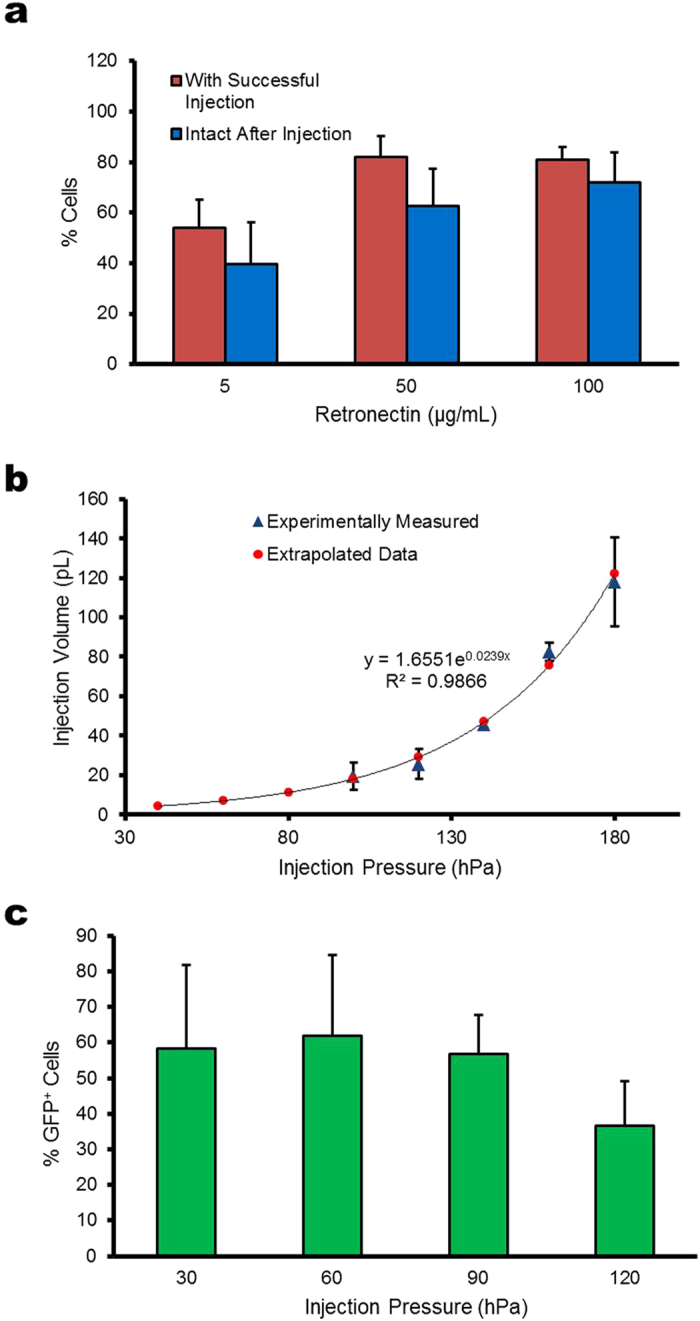
Characterization of microinjection into K562 cells. (**a**) Percentage of successfully injected (red bars) and cells intact after injection (blue bars) on polystyrene surfaces coated with solutions containing different initial concentrations of retronectin. Intact cells are defined as retaining dextran fluorescence 2 hours after injection. Each experiment consisted of 30–40 cells injected at each initial coating concentration. Bars represent the mean of 4 independent experiments ± the standard deviation. (**b**) Plot of injection volume for different injection pressure settings. The injection volume was estimated experimentally by performing injections of dye labeled dextran into a droplet of mineral oil. The injected sphere was estimated using the equation: 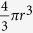, for pressure settings between 100–180 hPa, where 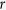 is the radius of the injected solution measured using a micrometer. The equation for the best fit exponential curve was used to estimate the injection volume for pressure settings below 100 hPa. (**c**) Plot of the percentage of injected cells with observable GFP expression at 24 hours after injection with different pressure settings. Bars represent the mean percentage of GFP expressing cells out of 35–56 cells injected ± the standard deviation for 4 independent experiments.

Microinjection into K562 cells using glass microcapillaries was facilitated by immobilizing cells on polystyrene surfaces coated with retronectin (Supplementary Figure 1a). The success of injection was assessed by supplementing the injection solution with FITC-dextran (10 kDa) as a fluorescence marker. Retronectin is recombinant human fibronectin consisting of a CS-1 site and RGDS domain that interact with the α_4_β_1_ and α_5_β_1_ integrins expressed on K562 cell membranes^36^. Injections were performed at ambient conditions at a rate of ~4 cells per minute with injection sessions limited to 40 minutes per plate to ensure high cell viability. Cells adhering to retronectin-coated surfaces had a flattened, spherical morphology and remained firmly bound following injection (Supplementary Figure 1a). We evaluated the effects of the cell-matrix interaction on cell viability by seeding and detaching cells on polystyrene surfaces coated with solutions containing different initial retronectin concentrations. Consistent with a previous study^21^, detachment of cells from retronectin coated surfaces after incubation and attachment for 1.5 hours did not significantly affect the cell viability, even at increasing concentrations of retronectin (Supplementary Figure 1b). However, the retronectin concentration had an effect on the attachment of cells under the force applied by the glass microcapillary during injection (Fig. 1a). We found that the highest level of successful injection (82%) was achieved with an initial retronectin concentration of 50 μg/mL or greater in the coating solution (Fig. 1a). Cells immobilized on retronectin-coated surfaces had a cell viability of ~63–72% after injection (Fig. 1a), which was estimated by the percentage of cells that retained the dextran fluorescence and maintained membrane integrity after a 2 hour incubation period following injection. We used an initial retronectin concentration of 50 or 100 μg/mL in the coating solution for all injections in this study.

### Injection parameter optimization

We used a FemtoJet (Eppendorf) microinjector to control the volume released by adjusting the applied injection pressure and duration of injection. To generate a standard curve correlating injection pressure and volume, we injected dye-labeled dextran into mineral oil using different injection pressures and fixed injection time of 0.1 seconds and measured the diameter of the injected spheres using a micrometer^37^. The results obtained (Fig. 1b) are consistent with the volume vs pressure measurements reported in a study^38^ that used a different approach for estimating the injection volume. To determine the optimal injection pressure, cells immobilized on a retronectin-coated surface were co-injected with TRITC-dextran (10 kDa), a fluorescence reporter for injection, and pmaxGFP plasmid DNA with different injection pressures ranging from 30 to 120 hPa (corresponding to injection volumes between 3 to 30 pL). At 24 hours after injection, signal from dye-labeled dextran was used to identify the injected cells^30^ (Supplementary Figure 2a) and the percentage of injected cells that exhibited GFP fluorescence was quantified^39^ (Fig. 1c). We found that an injection volume of 7 pL gave the highest amount of GFP-expressing cells (Fig. 1c). Injection volumes greater than 30 pL resulted in noticeable cell damage and cell death. An example of a K562 cell with membrane damage induced by microinjection is shown in Supplementary Figure 2b. Thus, an injection volume of 7 pL was used for all subsequent microinjection experiments.

### The effects of microinjection on cell doubling time

The effect of glass microcapillary injection on cell doubling time was evaluated. We focus on examining the potential cytotoxic effect due to microinjection only by injecting pmaxGFP plasmid instead of nuclease-encoding plasmid, since nucleases can have cytotoxic effects in cells partly due to off-target cleavage activity. Cells successfully injected with FITC-dextran or nucleofected with pmaxGFP plasmid (as a positive control) at 24 hours after delivery were deposited into Terasaki MicroWell plates at 1 cell per well using fluorescence activated cell sorting (FACS) (Supplementary Figure 3) and subsequently cultured for two days. Non-treated cells detached from retronectin-coated plates or cultured in suspension on uncoated plates were used as controls. The cell number in each well was counted at 24 and 48 hours after sorting and then used to calculate the cell doubling time. We estimated the doubling time for control cells to be 20–23 hours (Supplementary Figure 4), which is similar to the doubling rate reported in the literature^40^. Consistent with that shown previously^21^, the microinjection method had no adverse effect on the cell doubling time, and nucleofection did not have much effect on the cell doubling rate either (Supplementary Figure 4).

### Controlled delivery of *HBB*-targeting nucleases using microinjection

**Figure 2:**
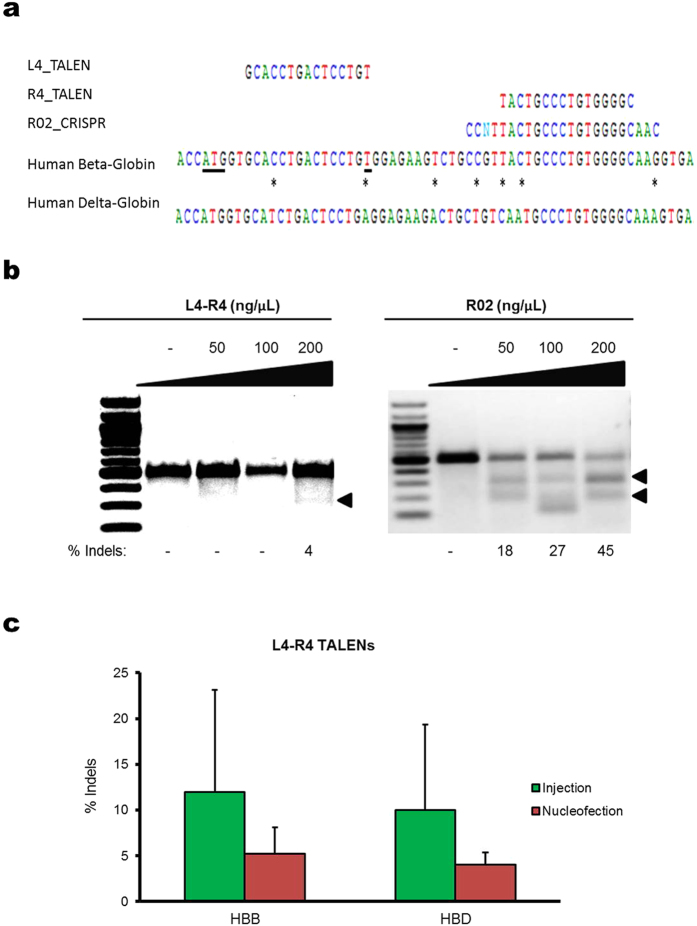
Gene editing by *HBB*-targeting nucleases using microinjection. (**a**) Schematic showing targeting sequences of L4-R4 TALEN pair and R02 CRISPR guide RNA aligned to *HBB* and *HBD*. The CRISPR guide RNA is shown complementary to the reverse strand and is listed to the right of the PAM sequence. The ATG start codon and the sickle cell mutation are underlined. Asterisks between *HBB* and *HBD* indicate mismatches. The A, T, C, and G nucleotides are shown in green, red, blue, and black respectively for clarity. (**b**) Nuclease-induced indel rate as a function of plasmid concentration. Plasmids encoding L4-R4 TALENs or R02 CRISPR/Cas9 were microinjected into K562 cells with an injection volume of 7 pL and the nuclease-induced cleavage at *HBB* was analyzed using the T7E1 assay. Shown is a comparison of the indel rates by L4-R4 TALENs and R02 CRISPR/Cas9 system at plasmid concentrations of 50, 100 and 200 ng/μL. **(c)** Comparison of indel rates at *HBB* and *HBD* induced by the L4-R4 TALEN pair delivered using microinjection and nucleofection. Green and red bars represent mean percent indels in microinjected and nucleofected cells respectively. The indels shown for microinjected cells represent the average of 58 single cell clones pooled together per sample. N = 3 for cells microinjected and nucleofected with L4-R4 TALENs.

Microinjection enables simultaneous delivery of multiple genome editing reagents, including nucleases and donor template DNA, into cells. To demonstrate the ability of microinjection-based delivery for genome editing, K562 cells were injected with plasmids expressing TALENs or CRISPR/Cas9 respectively, and the nuclease on- and off-target activities were quantified. The TALEN pair L4 (left TALEN) and R4 (right TALEN) used in this study was designed to target the beta globin gene^9^, the target sites are shown in Fig. 2a. The L4-TALEN overlaps the sickle mutation in codon 6 of *HBB*, separated by a 15-base spacer from the R4-TALEN binding site (Fig. 2a). Note that cells with wild-type *HBB* were used in this study. The CRISPR R02, a 20-base guide sequence, was designed to target *HBB* as well^11^, near the sickle mutation (Fig. 2a) adjacent to a PAM sequence containing the trinucleotide NGG. To label injected cells, in addition to plasmids encoding TALENs or CRISPR/Cas9, K562 cells were co-injected with FITC-dextran as a fluorescence marker.

Successfully injected cells were deposited into 96-well plates with 1 cell per well on average using FACS. The clonal colonies derived from the single microinjected cells after 14–16 days of culturing were pooled together. The T7E1 mutation detection assay was performed to quantify the rate of cleavage-induced insertions and deletions (indels). We found that the on-target cleavage rate is dose-dependent and, for the L4-R4 TALEN pair tested, the indel rate was 4% at a concentration of 200 ng/μL total TALEN plasmid, while no measurable activity at concentrations of 50 and 100 ng/μL was observed (Fig. 2b). In contrast, for the CRISPR/Cas9 system tested, much higher indel rates were obtained (Fig. 2b). Specifically, with plasmid encoding R02 CRISPR/Cas9, indel rates of 18%, 27%, 45% were obtained at plasmid concentrations of 50, 100 and 200 ng/μL respectively.

To benchmark the cleavage activity measured in the microinjection studies, we compared the on- and off-target activity in K562 cells nucleofected with L4-R4 TALENs. Cells were nucleofected with plasmids encoding L4-R4 TALENs using a 4D-nucleofector system (Lonza) and cultured for 3-days following nucleofection. The T7E1 assay was performed to measure the L4-R4 TALEN induced indels at *HBB* in bulk nucleofected and microinjected cells (Supplementary Figure 5). Off-target activity was measured at *HBD*, which has a sequence similar to the *HBB* target site (Fig. 2a). Interestingly, the mean cleavage activity in microinjected cells was slightly higher compared to nucleofected cells, although the difference was not significant (Fig. 2c). This indicates that the L4-R4 TALENs expressed in cells following microinjection are highly active, providing further evidence that microinjection works well for delivering genome editing reagents into human somatic cells.

### Single-cell analysis of on- and off-target indels

**Figure 3:**
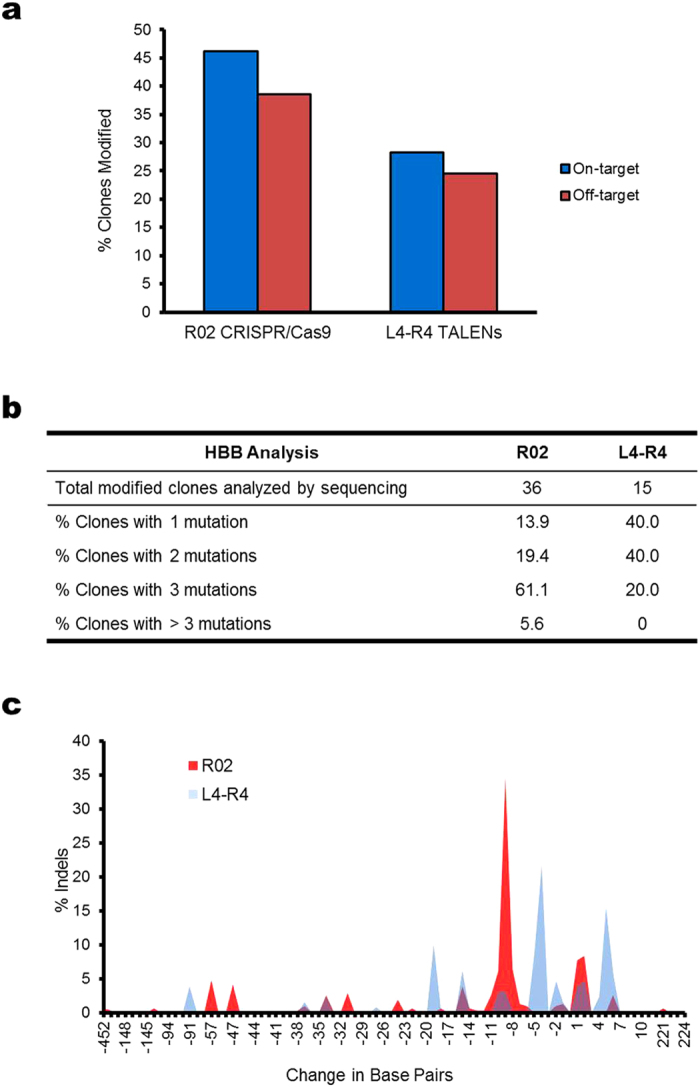
On- and off-target indels in single K562 cells microinjected with *HBB*-targeting nucleases. Cells microinjected with 200 ng/μL plasmids encoding L4-R4 TALENs or R02 CRISPR/Cas9 were expanded from single cells into clonal colonies in a 14 to16-day culture. T7E1 assay and Sanger sequencing were performed in individual clones and the number of clones having measurable indels was determined. (**a**) The percentages of clones with measurable on- and off-target indels. The off-target indels were detected in the *GRIN3A* locus and *HBD* for R02 CRISPR/Cas9 and L4-R4 TALENs respectively. N = 78 (R02) and 53 (L4-R4). (**b**) The percentage of clones having specific numbers of *HBB* mutations detected from 8–24 total sequencing reads. (**c**) Indel spectrum in cells microinjected with L4-R4 TALENs or R02 CRISPR/Cas9 determined using Sanger sequencing. The change in the number of base pairs resulting from NHEJ repair of DNA cleavage at the *HBB* target locus was compiled for each sequence read. The y-axis represents the percentage of indels with specified number of base pair changes.

We performed single-cell analysis of indels in cells microinjected with the R02 CRISPR/Cas9 system and L4-R4 TALENs respectively. Clonal colonies were generated from K562 cells microinjected with nucleases by depositing single injected cells into multi-well plates (one cell per well) using FACS, followed by culturing for 14 to 16-days. On- and off-target activities of clones derived from single microinjected cells were measured using the T7E1 assay and the percentage of clones with indels was determined. For the R02 CRISPR/Cas9 system, off-target cleavage was evaluated at the *GRIN3A* locus, which was shown to have a high level of off-target cleavage^11^. We found that with the R02 CRISPR/Cas9 system, 36 out of 78 clones (46%) had modifications at *HBB* while 30 clones (38.5%) showed indels at the *GRIN3A* locus (Fig. 3a). In contrast, for the 53 clones derived from single cells injected with L4-R4 TALENs, 28% on-target (*HBB*) and 24.5% off-target (*HBD*) indels were found (Fig. 3a). Compared with bulk measurements, these results may give a more accurate quantitation of the cleavage efficiencies for CRISPR/Cas9 and TALENs, since the amount of nuclease-encoding plasmids delivered is more uniform among the cells.

To gain additional insight into nuclease-induced DNA cleavage, the clones having measurable indel rates were further analyzed using Sanger sequencing. PCR primers used to amplify the *HBB* target region for sequencing were designed with a 5′ 4-base barcode (Supplementary Table 3) so that each clone had a unique sequence identifier. The percentage of clones that had 1, 2, 3 or >3 mutations in *HBB* was quantified. We found that, of the 36 clones having R02 CRISPR/Cas9 induced cleavage at *HBB*, 22 (61.1%) had 3 *HBB* mutations (Fig. 3b), indicating a high level of Cas9 activity with the target site cleaved in all three copies of chromosome 11^41^ (Fig. 3a). The capacity of the R02 CRISPR/Cas9 to provide high levels of triallelic cuts is a strong indicator of an efficient nuclease design. Consistent with results shown in Figs 2b and 3a, L4-R4 TALENs had lower cleavage activity compared to the R02 CRISPR/Cas9 system, with only 20% of the clones having 3 *HBB* mutations (Fig. 3b). Although single cells were sorted into the majority of wells (87%), single cell clones were not strictly guaranteed by the FACS equipment, such that roughly 3.4% of wells contain 2 or 3 cells following FACS (Supplementary Figure 6). The percentage of clones having more than 3 *HBB* mutations (Fig. 3b) was very similar to the expected frequency of wells containing more than 1 cell (Supplementary Figure 6). Therefore, it is likely that the observation of more than 3 *HBB* mutations resulted from multiple cell clones that were FACS sorted into the same well.

We further analyzed the indel spectra induced by R02 CRISPR/Cas9 and L4-R4 TALENs respectively. We found that cells injected with TALENs had a broad spectrum of indels, including peaks of 20-base and 4-base deletions and 5-base insertions (Fig. 3c). Similarly, the CRISPR/Cas9 system induced a broad spectrum of indels as well, with sharp peaks of 9-base deletions and a 1-base insertion (Fig. 3c). The observation that 9-base deletion is the most frequent indel in cells injected with R02 CRISPR/Cas9 is consistent with that for cells subjected to nucleofection (Supplementary Figure 7). However, cells nucleofected with R02 CRISPR/Cas9 had a peak of 5-base deletions that was absent in the microinjection data, which is likely because a considerably larger number of cells were used for obtaining DNA sequencing in the nucleofected sample. The difference in indel spectra between CRISPR/Cas9 and TALENs is likely due to the specific DNA cleavage induced (blunt ends vs. 4-base overhang) and the corresponding repair mechanism.

### Quantifying HDR-mediated gene modification in microinjected cells

**Figure 4:**
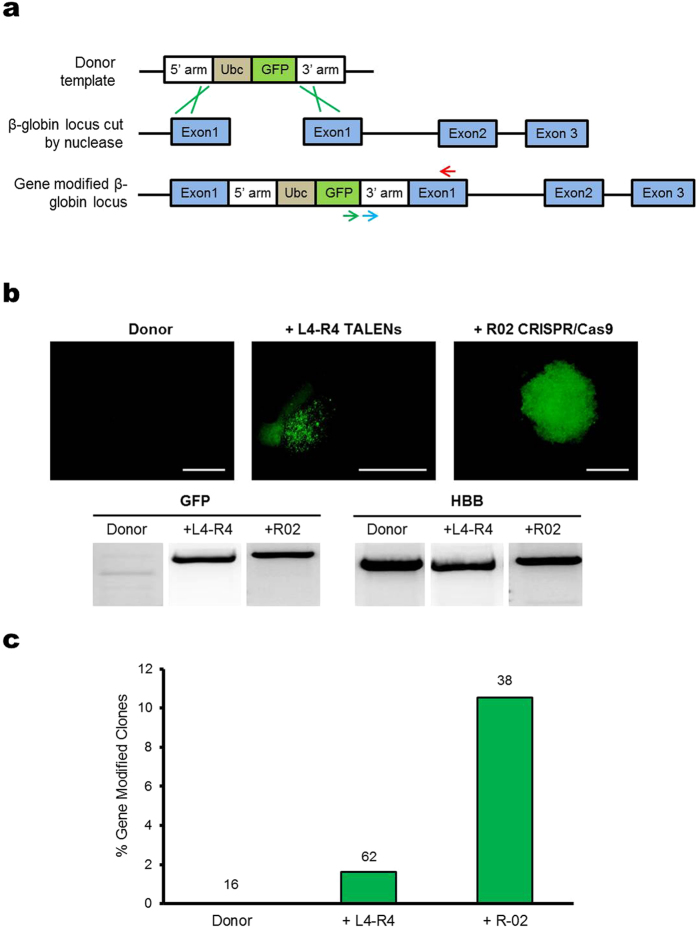
HDR-mediated gene modification in microinjected K562 cells. (**a**) Diagram of GFP reporter system used to detect HDR. Nuclease cleavage and resection yields a substrate for HDR which may involve the use of exogenous β-Ubc-GFP donor, flanked by 5′ and 3′ homologous fragments of *HBB* sequence (top). The green lines indicate the HDR process with the donor template. Gene insertion was confirmed by PCR using primers for integrated GFP at the target locus, as shown by the green and red arrows (bottom). The *HBB* gene (control) was amplified using primers shown by the blue and red arrows, which bound downstream the 3′ homologous region in *HBB*. (**b**) Fluorescence microscopy images of clones derived from single cells microinjected with β-Ubc-GFP donor with or without L4-R4 TALENs or R02 CRISPR/Cas9. Bottom of images are PCR results of integrated GFP or *HBB* (control) for clones. Scale bar corresponds to 500 μm. (**c**) The percentage of clones with HDR-mediated gene modification confirmed by both PCR and fluorescence microscopy. The number of single cell clones analyzed is shown above each bar.

We also investigated HDR-mediated gene modification efficiency for the L4-R4 TALENs and R02 CRISPR/Cas9 system in cells microinjected using glass microcapillaries. The donor template for targeted gene insertion was designed with a GFP expression cassette under the Ubc promoter and flanked by approximately 1 kb arms of homology from the *HBB* locus^9^ (Fig. 4a). Primers listed in Supplementary Table 2 were designed to amplify the integrated GFP sequence in the *HBB* locus. We co-injected the β-Ubc-GFP donor template with L4-R4 TALENs or R02 CRISPR/Cas9 into cells, isolated injected cells using cell sorting and formed single cell colonies in a 14 to 16-day culture. Clones were evaluated for GFP expression using fluorescence microscopy. We found that many clones from cells co-injected with *HBB*-targeting nucleases and β-Ubc-GFP donor were positive for GFP fluorescence, but not the case for clones formed with cells injected with donor only (Fig. 4b). To confirm targeted gene insertion, we extracted the genomic DNA from cells showing GFP fluorescence, and PCR amplified the GFP sequence integrated at the *HBB* site (Fig. 4b). We defined clones with HDR-mediated gene modification as having both GFP fluorescence and positive PCR results. To quantify the efficiency of HDR, we determined the number of clones with gene modification, divided by the total number of clones derived from cells injected with both nuclease and donor. This method of quantifying the HDR rate is more rigorous compared to flow cytometry analysis of GFP fluorescence alone^9^ because of the additional requirement for PCR detection of GFP integration at the *HBB* target site. Of the 38 clones derived from cells co-injected with R02 CRISPR/Cas9 and donor template, we observed 4 clones (10.5%) with PCR-confirmed gene modification (Fig. 4c). In contrast, for clones derived from cells co-injected with L4-R4 TALENs and donor template, only 1.6% had PCR-confirmed GFP gene modification (Fig. 4c).

**Figure 5:**
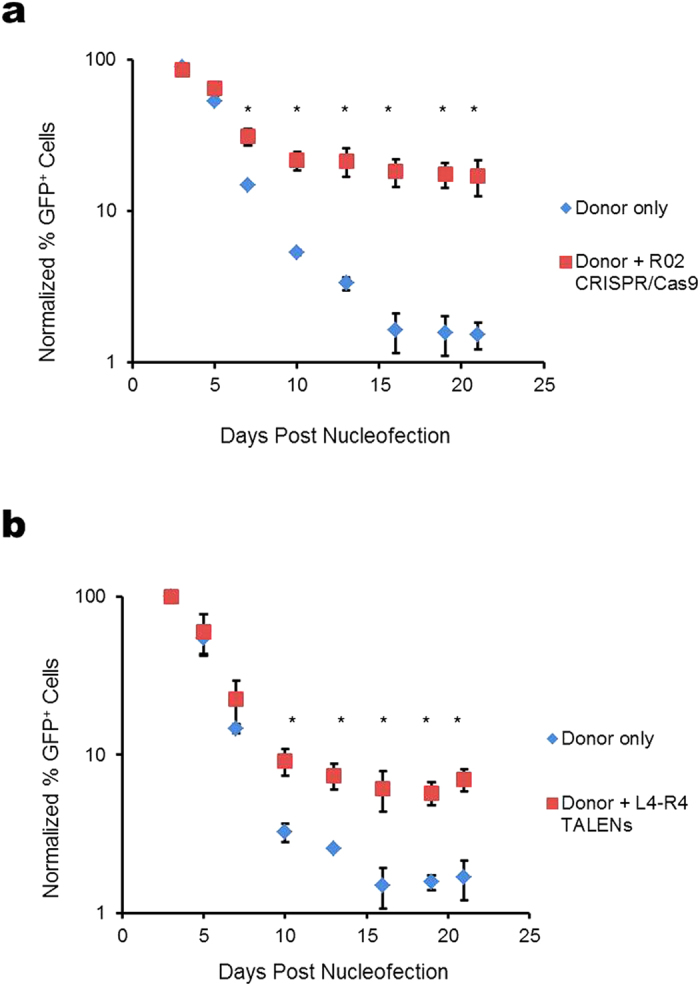
Gene insertion in nucleofected cells. The percentage of GFP-positive cells was quantified using flow cytometry during a 21-day culture after nucleofection with β-Ubc-GFP donor with and without (**a**) R02 CRISPR/Cas9 or (**b**) L4-R4 TALENs. The plots show normalized percentage of GFP-positive cells at specified days post nucleofection. Asterisks indicate significant difference between donor only and donor plus nuclease at specified days.

To quantify and compare the frequency of HDR-mediated gene modification using microinjection and nucleofection respectively, cells were nucleofected with the β-Ubc-GFP donor together with *HBB* L4-R4 TALENs or R02 CRISPR/Cas9 and cultured in bulk for up to 21 days. Cells were then analyzed using flow cytometry to obtain the percentage of cells having gene-insertion induced GFP fluorescence, and the results were normalized using the Day 3 results. We found that for cells nucleofected with R02 CRISPR/Cas9 and donor template, there was an 11-fold increase in the normalized amount of GFP-positive cells compared to cells with donor only (Fig. 5a). In contrast, there was only a 4-fold increase for cells nucleofected with L4-R4 TALENs plus donor (Fig. 5b). The higher amount of GFP-positive cells due to CRISPR/Cas9 induced gene insertion compared to that of TALENs is consistent with the microinjection results (Fig. 4c).

## Discussion

In this work we systematically characterized a microinjection-based method for the direct delivery of genome editing reagents into human K562 cells. Similar to that reported previously^21^, we found that adhesion of suspension cells to a surface coated with a sufficient concentration of retronectin facilitates microinjection (Fig. 1a). We demonstrated the ability of using FACS to generate clones from single injected cells for the analysis of on- and off-target cleavage rates of different nucleases. The results from this study indicate that the glass microcapillary-mediated microinjection method does not adversely affect the proliferation potential of cells (Supplementary Figure 4). Furthermore, the measured cell doubling time suggests that the cytotoxicity from the microinjection method is similar to that with nucleofection (Supplementary Figure 4). Using the microinjection method, we demonstrated high levels of targeted indels (Fig. 3) and gene modification in human somatic cells by TALENs and CRISPR/Cas9 systems (Fig. 4c). In our study, the amount of plasmids injected into single cells gave on-target cleavage rates similar to that with nucleofection. However, it can be further optimized to have reduced off-target activity.

Compared with other delivery methods for genome editing, such as cationic lipid transfection, nucleofection, viral-based delivery and receptor-mediated protein uptake^42^, microinjection has potential advantages, including: (1) precise control of the amount delivered into single cells, (2) applicable to a wide range of cell types, (3) robust in delivering different forms of genome editing reagents (DNA, RNA, protein) and (4) with minimal waste of reagents^35^. Given that nucleases have cytotoxicity, at least partly, due to their activity at off-target sites^13^,^14^,^15^, precise control over the amount of nucleases injected into single cells may be useful to minimize the off-target effects, while maintaining a high level of on-target cleavage. Further, the delivery of TALEN proteins or CRISPR/Cas9 ribonucleoproteins using lipofection^43^, electroporation^44^, cell-penetrating peptides^45^,^46^,^47^, and lentiviral vectors^48^ has been shown to reduce the off-target effects relative to plasmid DNA delivery. Because microinjection is amenable to protein^24^,^31^,^49^ and RNA^50^,^51^,^52^ delivery, it is feasible to inject nucleases as mRNAs or purified proteins into single cells using the approach described in our study, which may lead to optimal ratios of off-target/on-target cleavage activities compared to plasmid DNA.

Although microinjection has been well established for cellular delivery^20^,^21^,^22^,^23^,^24^,^25^,^26^,^27^,^28^,^29^,^30^,^31^, our study is the first to demonstrate its use for delivering gene editing reagents into human somatic cells and achieve a high level of gene modification, including gene insertion. However, although we were able to microinject up to 1000 cells on retronectin coated dishes within 4–6 hours, the microinjection-based approach is low throughput, which is a major limitation. Clearly, there is a need to develop high throughput microinjection systems, especially for applications where processing a large number of cells is required.

## Materials and Methods

### *HBB*-targeting nucleases and donor constructs

The *HBB* NN TALEN (L4-R4) plasmids and β-Ubc-GFP donor template constructs described in^9^ were gifts from Dr. Matthew Porteus at Stanford University. The heterodimeric L4- and R4-TALEN target sequences (underlined) separated by a 15-base spacer region: 5′-GCACCTGACTCCTG**T**GGAGAAGTCTGCCGTTACTGCCCT GTGGGG C-3′. The 20-base target sequence (underlined) following the 3-base PAM for the *HBB*-aiming CRISPR (R02) construct described in^11^: 5′-G**T**GGAGAAGTCTGCCGTTACTGCCCTGTGGGGCAAC-3′. The nuclease target sites overlap or are in close proximity to the sickle cell mutation (bold) in codon 6 of *HBB*.

### K562 cell culture conditions and nucleofection

K562 cells (ATCC, Manassas, VA) were grown in Iscove’s Modified Dulbecco’s Media (IMDM) supplemented with 10% FBS, 2 mM L-glutamine, and 1X penicillin/streptomycin supplement (Invitrogen Life Technologies, Grand Island, NY). For nucleofections, K562 cells were seeded at 1 × 10^6^ cells per well in 6-well dishes. The next day, 1 × 10^6^ or 2 × 10^5^ cells per reaction were nucleofected with specified constructs along with 100 ng pmaxGFP using SF cell line 4D-Nucleofection kit (Lonza, Walkersville, MD) according to the manufacturer’s protocol in biological triplicates. At 24 hours after nucleofection, the growth medium was changed in each well. The nucleofection efficiency was determined as the percentage of GFP expressing cells at 3 days after nucleofection using an Accuri C6 flow cytometer (BD Biosciences, Franklin Lakes, NJ). Suspension cultured K562 cells and all derivative clones were grown and maintained in the media conditions listed above in a humidity-controlled incubator with 5% CO_2_ at 37 °C.

### Glass microcapillary mediated-microinjection

Polystyrene dishes coated overnight at 4 °C with 50 or 100 μg/mL of human recombinant fibronectin fragment CH-296 (Retronectin; TAKARA BIO, Madison, WI) in PBS and washed with 2% BSA. Cells were seeded and attached to the retronectin coated dishes by incubating for roughly 2 hours at 37 °C. Cells were detached from the dishes by gentle pippetting. The percentage of viable cells detached from the coated surface was calculated using trypan blue staining and automated cell counter (BIO-RAD Laboratories, Hercules, CA).

Sterile glass microcapillaries with 0.5 μm inner tip diameter (Femtotips Narrow; Eppendorf, Hamburg, Germany) were assembled to a capillary holder. The microcapillary position and cell injection was controlled using a programmable InjectMan NI 2 micromanipulator (Eppendorf). Cells were visualized by phase and fluorescence microscopy using a Delta Vision Microscope system equipped with a computer-controlled stage. The pressure applied to release the injection solution from the microcapillary was supplied by a FemtoJet injector (Eppendorf) with built-in air compressor and programmable injection pressure settings to ensure reproducible injections. The pressure settings applied for cell injections: injection pressure of 60 hPa, injection time of 0.1 seconds, and counter-pressure of 30 hPa. Injections were performed at room temperature. Cells attached to retronectin coated dishes were visualized by phase contrast microscopy. The microcapillary tips were lowered over the cells until both were in the same focal plane, the injection level was defined and programmed into the semiautomatic micromanipulator, which controlled the injection movement at a 45° angle. Injected cells were assessed by fluorescence microscopy to verify successfully injected cells immediately after injection. The percentage of successfully injected cells was determined as the number of fluorescent cells divided by the number of cells injected × 100. The percentage of intact cells was determined as the number of fluorescent cells at 2 hours after injection divided by the number of fluorescent cells immediately after injection × 100. The volume released by the microcapillary was estimated by performing injections using various injection pressures into mineral oil droplets on a microscope slide using the protocol described in^37^. The injection volume was calculated using the equation: V = 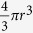, where V is the volume and 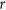 is the radius of the injected liquid, forming a sphere within the oil droplet. We found the injection volume has an exponential dependence on pressure and is consistent with predictions made in^53^.

### Preparation of microinjection solutions

Injection solutions were prepared in sterile, cold PBS. 10,000 Da dextran-Alexa Fluor 488 (FITC-dextran; Invitrogen) or 10,000 Da dextran-Alexa Fluor 594 (TRITC-dextran; Invitrogen) were adjusted to 1 mg/mL. Unless specified, pmaxGFP was adjusted to 200 ng/μL, L4-R4-TALEN plasmids were adjusted to 200 ng/μl, R02 CRISPR/Cas9 plasmid was adjusted to 200 ng/μL, and β-Ubc-GFP donor template vector was adjusted to 200 or 400 ng/μL. The pmaxGFP and β-Ubc-GFP donor template constructs contain GFP isolated from the jellyfish *Aequorea Victoria.* FITC-dextran was co-injected into cells along with L4-R4 TALENs or R02 CRISPR/Cas9 constructs as a marker for successful injection. Injection solutions were centrifuged at 13,000 × g for 20 min at 4 °C and the supernatant was directly loaded into microcapillaries for injection.

### Isolation of microinjected cells

Cells microinjected with solution supplemented with FITC-dextran were separated using a FACS Aria II system (BD Biosciences). Cells were gated according to FITC fluorescence intensity levels. For the cell doubling experiments shown in Supplementary Figure 4, cells were stained using To-pro3 (Invitrogen) and further gated for viable, injected or nucleofected cells. The precision mode of sort was set to single cell to ensure high purity. Injected or non-injected cells were deposited 1 cell per well directly into 96-well (Nunc; Thermo Fisher Scientific, Waltham, MA) or 72-well (Terasaki MicroWell; VWR International, Radnor, PA) tissue culture plates containing growth medium. Single injected cells were expanded into clones in a 14–16 day culture.

### T7E1 mismatch detection assay

Cleavage activity was quantified in pooled or individual clones derived from single cells microinjected with L4-R4 TALENs or R02 CRISPR/Cas9 expressing plasmids. The off-target activity was measured at *HBD* and *GRIN3A* loci for L4-R4 TALENs and R02 CRISPR/Cas9 respectively as in^9^,^11^. The genomic DNA from pelleted cells were processed for PCR amplification as described in^54^. The genomic DNA was harvested using QuickExtract DNA extraction solution (Epicenter Biotechnologies, Madison, WI) and subjected to PCR amplification of on- and off-target loci using primers listed in Supplementary Table 1. All PCR reactions in 50 μL volume consisting of 1.5 μl genomic DNA were performed using AccuPrime Taq DNA High Fidelity Polymerase kit (Invitrogen) according to the manufacturer’s protocol for 40 cycles (94 °C for 30 sec, 60 °C for 30 sec, 68 °C for 45 sec). 200 ng of PCR product supplemented with 1X Accuprime buffer II were processed using cycles of melting and re-annealing (95 °C for 10 min, 95–85 °C at −2 °C/s, 85-25 °C at −0.1 °C/s). T7E1 (New England Biolabs, Ipswich, MA) was added to a final concentration of 250 units/mL and incubated at 37 °C for 60 minutes for digestion of mismatch duplexes. Reactions were resolved on a 2% agarose Tris-acetate-EDTA gel stained with ethidium bromide and observed with a UV imaging station. The intensity of bands corresponding to cleaved and uncleaved PCR product was measured by densitometry analysis using ImageJ software. The percentage of indels was estimated using the equation: 100 × (1-(1-fraction cleaved)^1/2^) as described in^54^.

### Quantification of HDR-mediated gene modification

For microinjection experiments shown in Fig. 4, cells were microinjected with 2:1 ratio of L4-R4 TALENs to β-Ubc-GFP donor or 1:2 ratio of R02 CRISPR/Cas9 to β-Ubc-GFP donor. Successfully injected cells were FACS deposited as 1 cell per well into 96-well plates and cultured for 14–16 days in growth medium. GFP fluorescence was observed in single cell clones using fluorescence microscopy. Genomic DNA from individual clones that were positive for GFP fluorescence was harvested and subjected to PCR using *HBB* integrated GFP amplification primers listed in Supplementary Table 2. Cells with HDR-mediated gene modification were defined as having both GFP fluorescence and PCR amplified integrated GFP.

In nucleofection experiments shown in Fig. 5, cells (1 × 10^6^) were seeded into 6-well plates and nucleofected with 2 μg R02 CRISPR/Cas9 or L4-R4 TALENs with and without 10 μg β-Ubc-GFP donor plasmid. pUC18 (Mock) plasmid was added to bring the total DNA amount to 12 μg for each reaction. Cells were incubated in growth medium for 21-days and the percentage of GFP cells was analyzed using an Accuri C6 flow cytometer at specified time points. The percentage of GFP-positive cells was normalized using Day 3 data.

### Sequencing for on-target indel rates

Individual clones derived from single cells microinjected with L4-R4 TALENs or R02 CRISPR/Cas9 were PCR amplified using barcoded *HBB* primers listed in Supplementary Table 3. PCR amplicons from clones having positive on-target T7E1 results were separately ligated into a vector and transformed into competent cells using the NEB PCR Cloning kit (New England Biolabs) or Zero Blunt TOPO Cloning kit (Invitrogen) according to the manufacturers’ protocols. Plasmid DNA from picked *E. coli* colonies were purified and sequenced using Sanger DNA sequencing technology (Operon, Huntsville, AL). Barcodes ensured that sequences were properly matched to individual clones derived from single microinjected cells.

For K562 cells nucleofected with R02 CRISPR/Cas9, HBB alleles were PCR amplified as previously described^55^ and sequencing was performed on the PacBio RS (Pacific Biosciences). A custom SMRT sequencing analysis pipeline was used for data analysis^55^.

### Quantifying the efficiency of single cell sorting by FACS

To accurately quantify the sorting efficiency for the FACS equipment, we used Lin^-^Sca-1^+^kit^+^ cells from the bone marrow of a 10 week old C57BL/6-TG(CAG-EGFP)10sb/J mouse stained as described in^56^. Lin^-^Sca-1^+^kit^+^ cells were single cell sorted into 72-well (Terasaki MicroWell) dishes containing 18 μL of α-MEM (Invitrogen) using a BD FACS Aria II. Immediately after cell sorting, the plates were viewed using a fluorescence microscope and the number of cells in each well was counted. We quantified the percentage of wells that contained 0, 1, or 2 or more cells. The data from this experiment is presented in Supplementary Figure 6.

### Statistical analysis

Significance was determined from three or more replicates or samples using Student’s t-test or one-way ANOVA. P-values < 0.05 were considered statistically significant. Bars or data points in plots are shown as statistical mean ± standard deviation.

## Additional Information

**How to cite this article**: Cottle, R. N. *et al.* Controlled delivery of β-globin-targeting TALENs and CRISPR/Cas9 into mammalian cells for genome editing using microinjection. *Sci. Rep.* **5**, 16031; doi: 10.1038/srep16031 (2015).

## Supplementary Material

Supplementary Information
