# Supplementary material for: Controlled delivery of β-globin-targeting TALENs and CRISPR/Cas9 into mammalian cells for genome editing using microinjection
Source: Sci Rep. 2015 Nov 12;5:16031. doi: 10.1038/srep16031 (PMC4642230; doi:10.1038/srep16031)
Supplement: Supplementary Information [file srep16031-s1.pdf]

Controlled delivery of β-globin-targeting TALENs and CRISPR/Cas9 into mammalian cells for genome editing using microinjection

Renee N. Cottle, Ciaran M. Lee, David Archer, and Gang Bao

Supplementary Information Contents

Supplementary Figure 1. Microinjected K562 cells on retronectin coated polystyrene dishes.

Supplementary Figure 2. Gene expression efficiency in K562 cells.

Supplementary Figure 3. Separation of microinjected K562 cells using FACS.

Supplementary Figure 4. K562 cell doubling time after microinjection and nucleofection.

Supplementary Figure 5. T7E1 mutation detection assays for L4-R4 TALENs targeting *HBB* and off-target indels at *HBD*.

Supplementary Figure 6. Efficiency for sorting single cells by FACS.

Supplementary Figure 7. Indel spectrum in K562 cells nucleofected with R02 CRISPR/Cas9.

Supplementary Table 1. Sequences of primers used to amplify the endogenous genes for T7E1 mutation detection assays.

Supplementary Table 2. Sequences of primers used for PCR confirmation of HDR-mediated GFP integration in *HBB*.

Supplementary Table 3. Sequences of primers used for amplifying *HBB* in single cell clones for T7E1 assay and Sanger sequencing.

Supplementary Table 4. Analysis of clones with on- and off-target activity.

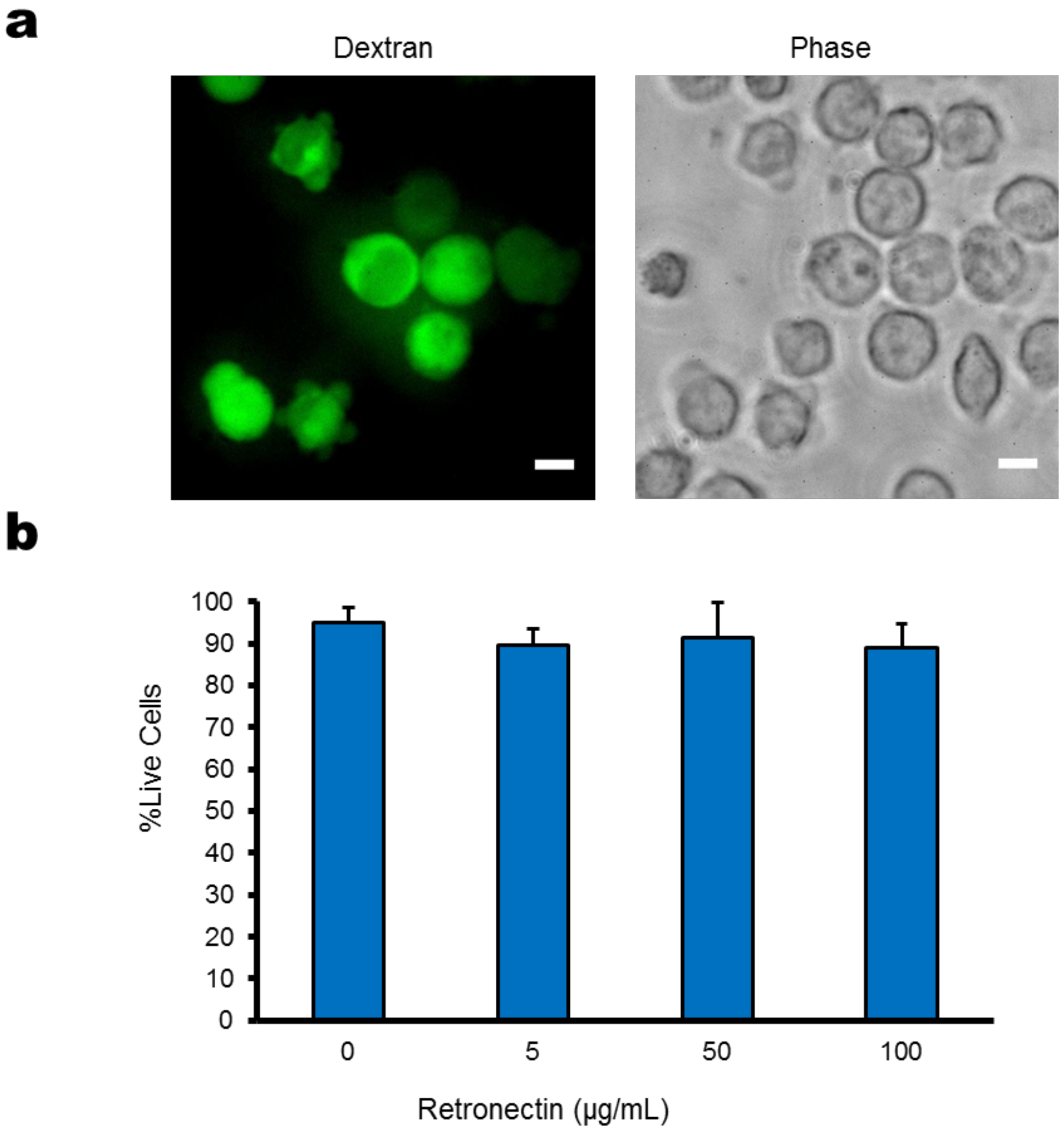

Supplementary Figure 1. Microinjected K562 cells on retronectin coated polystyrene dishes. **(a)** Fluorescence and phase contrast microscopy images of successfully injected cells immediately following injection with FITC-dextran. **(b)** Cell viability was not affected by retronectin as shown by the percentage of live cells detached from dishes coated with solutions containing different initial retronectin concentrations. For each sample, 3 X 10^4 cells attached to coated dishes were detached using pipetting. Bars represent statistical mean for 3 replicates ± the standard deviation. Scale bar width corresponds to 10 μm.

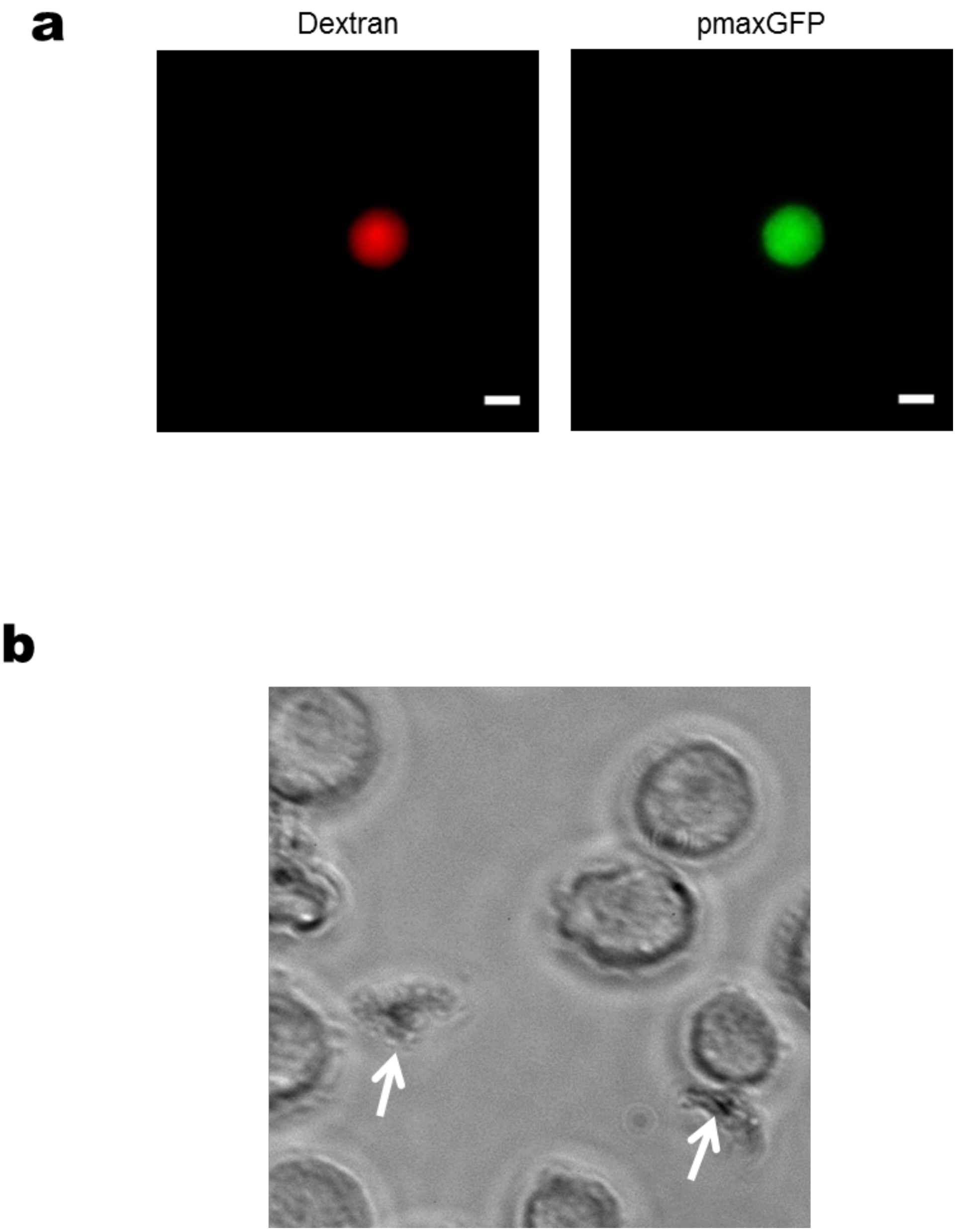

Supplementary Figure 2. Gene expression efficiency in K562 cells **(a)** Fluorescence microscopy images of K562 cells injected with TRITC-dextran and pmaxGFP plasmid at 24 hours after injection. Cells with successful injections (red) were analyzed for GFP expression (green). **(b)** Phase contrast image of K562 cells on a retronectin coated surface. The white arrows indicate the debris from a cell damaged by injection. Scale bar width corresponds to 10 μm.

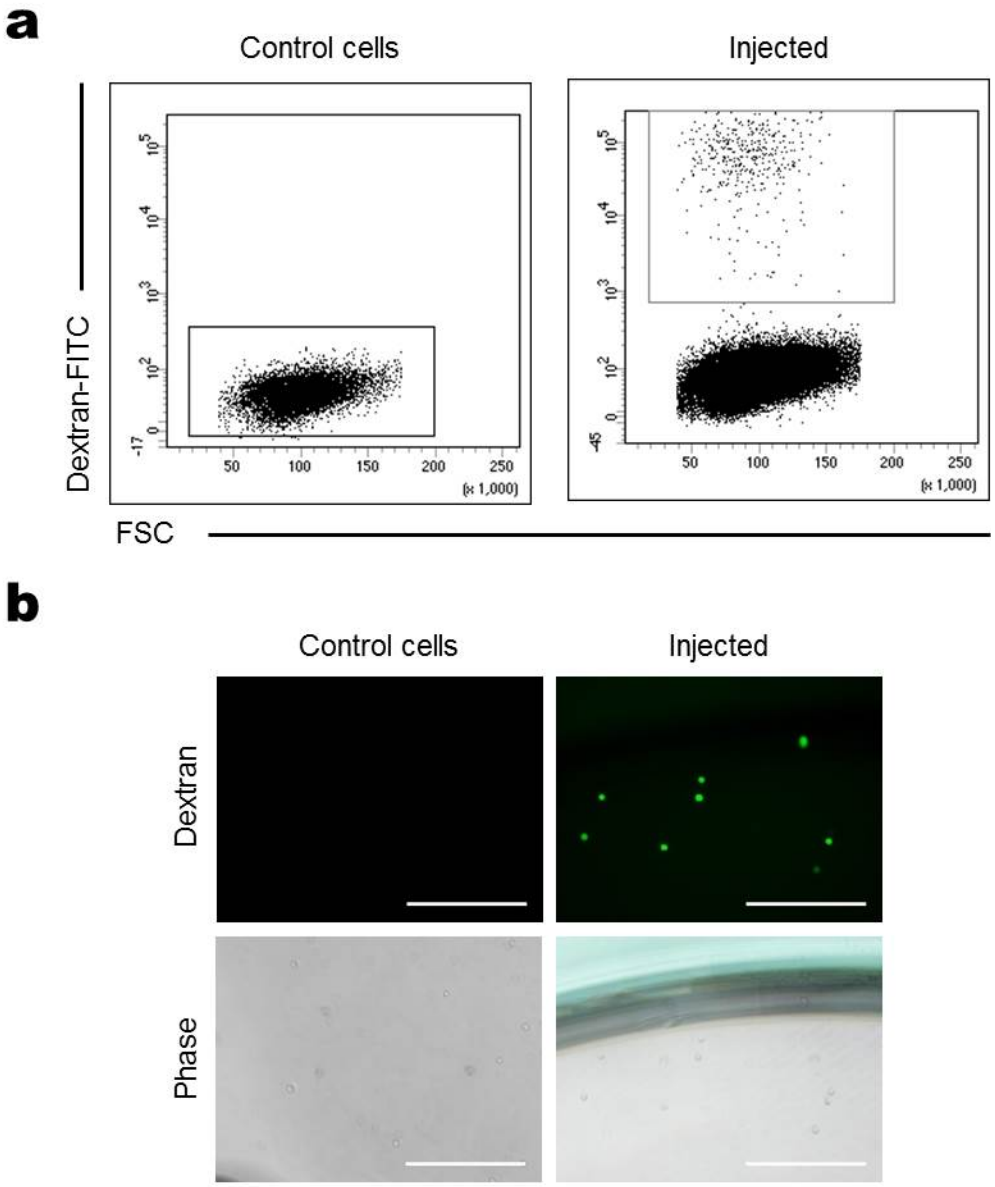

Supplementary Figure 3. Separation of microinjected K562 cells using FACS. Cells injected with FITC-dextran were detached from retronectin coated dishes after injection and subjected to FACS. **(a)** Panels show stable dextran fluorescence in cells gated for viability. For clarity, the control and injected K562 cells are shown in black and gray boxes respectively. **(b)** Fluorescence and phase microscopy images of injected and control cells after FACS. Scale bar width corresponds to 500 μm

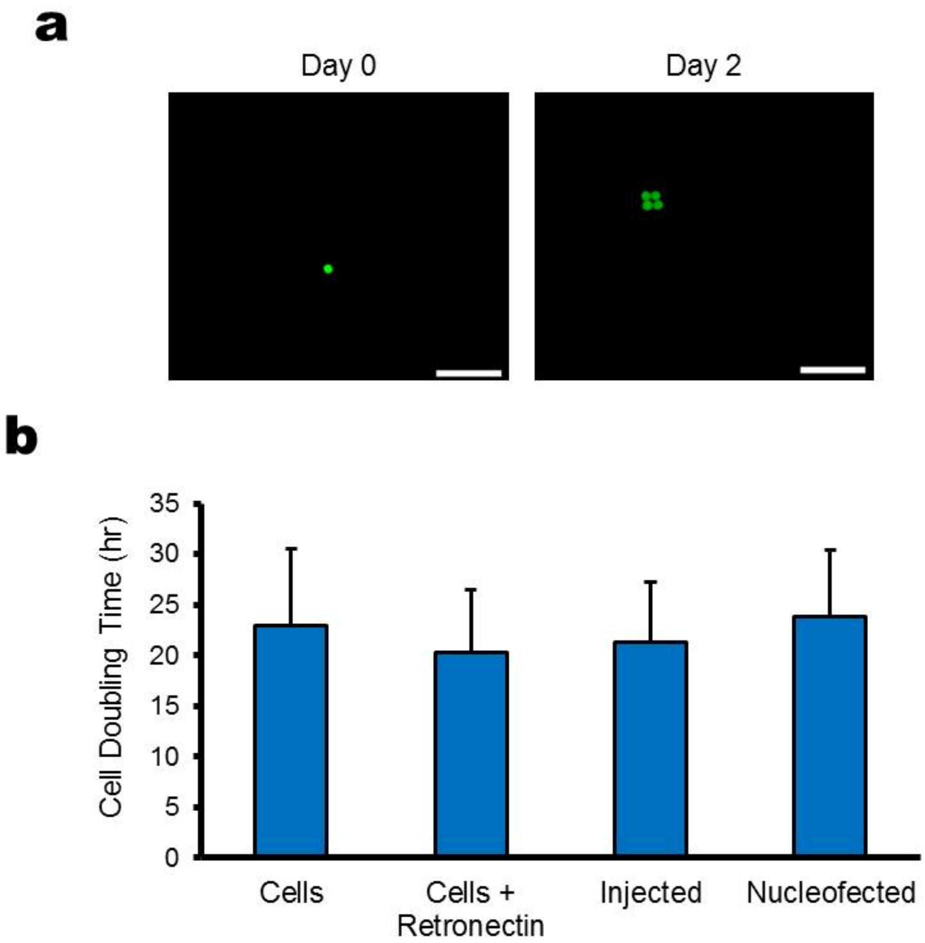

Supplementary Figure 4. K562 cell doubling time after microinjection and nucleofection. Viable K562 cells were deposited as single cells into Terasaki MicroWell plates using FACS at 24 hours after injection of FITC-dextran or nucleofection of pmaxGFP. **(a)** Fluorescence microscopy images of injected cells at 0 hours and 48 hours after FACS**. (b)** Plot of the cell doubling time for viable control and treated cells. The cell doubling time was calculated using the equation: incubation time between scoring cells x $\ln 2$ divided by $\ln \frac{x_e}{x_b}$, where x_e is the cell number at 48 hours and x_b is the cell number at 24 hours. As controls, we show the cell doubling time for viable untreated cells in suspension and detached from retronectin coated plates. One-way ANOVA indicates absence of a significant difference between different

conditions ($p > \alpha$, $\alpha = 0.05$). Scale bar width corresponds to 100 μm. Bars represent mean cell doubling time ± standard deviation (n = 3).

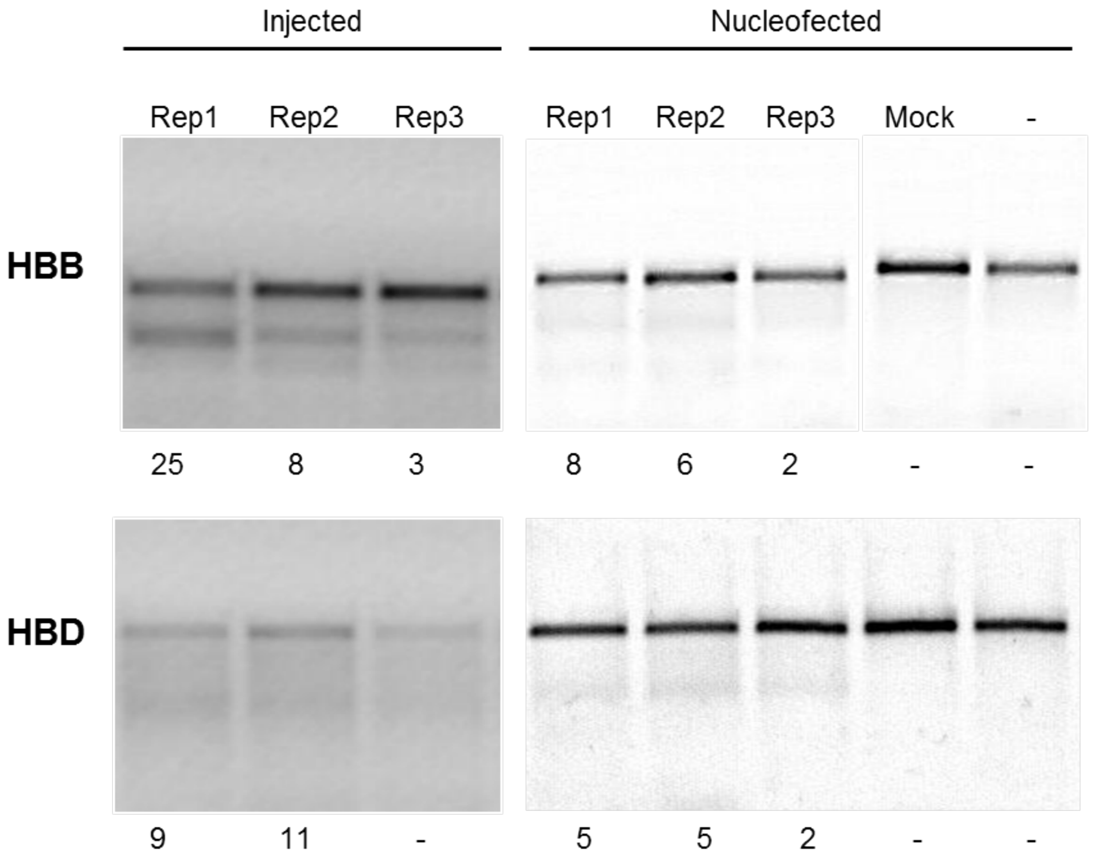

Supplementary Figure 5 T7E1 mutation detection assays for L4-R4 TALENs targeting *HBB* and off-target indels at *HBD*. K562 cells were microinjected or nucleofected with L4-R4 expressing plasmids. Experiments were repeated 3 times. The detectable indel percentages are shown below each lane.

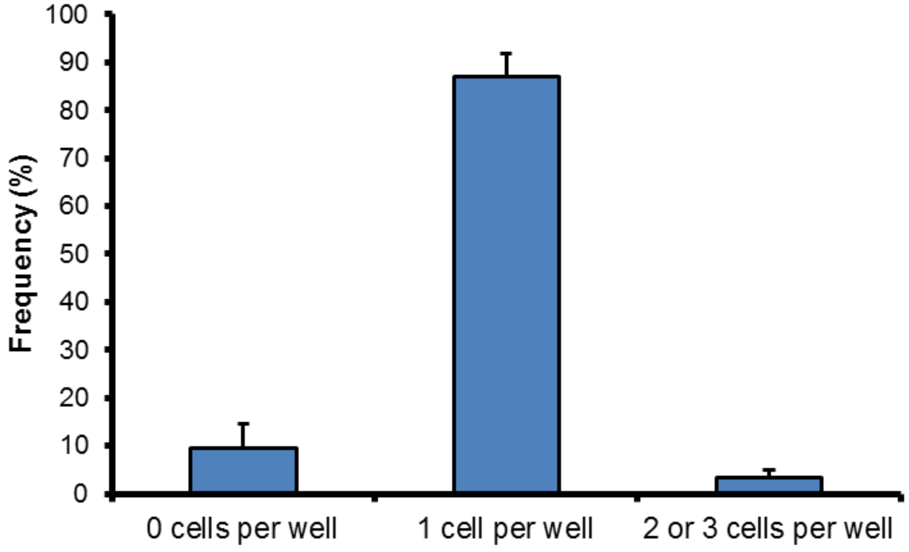

Supplementary Figure 6. Efficiency for sorting single cells by FACS. $eGFP^+$ $Lin^-Sca\text{-}1^+kit^+$ cells were single cell sorted into 66 wells of a Terasaki MicroWell dish. We counted the number of cells in each well using fluorescence microscopy. Up to 3 cells was counted for each well. The bars represent the mean percentage of wells that contained 0, 1, or 2 and more cells ± the standard deviation, n = 4.

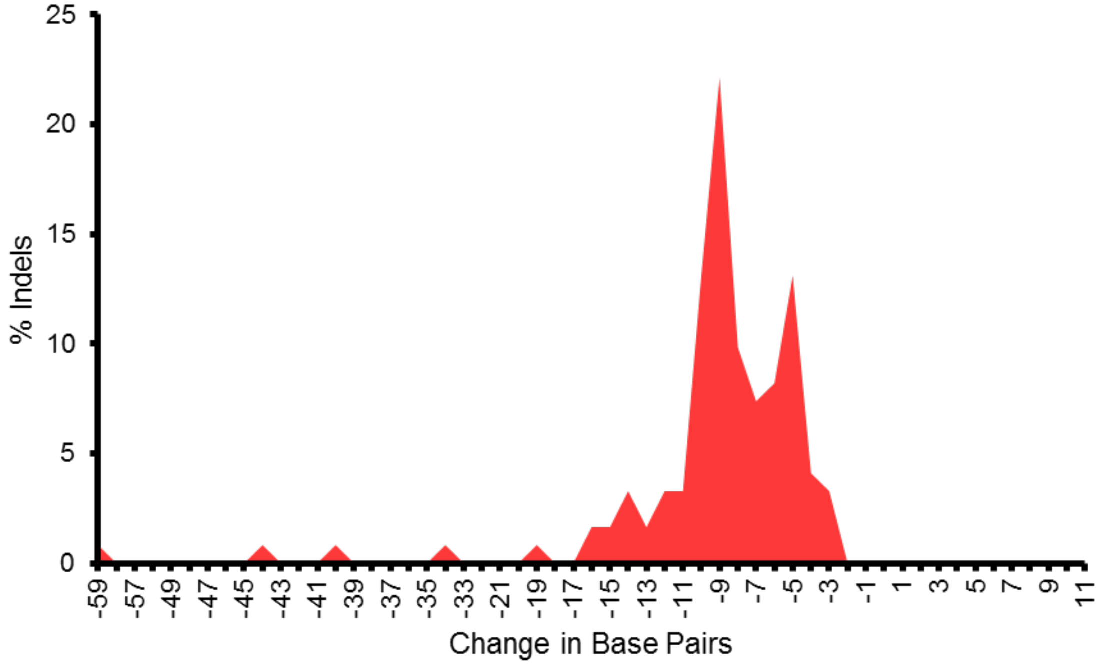

Supplementary Figure 7. Indel spectrum in K562 cells nucleofected with R02 CRISPR/Cas9. K562 cells (2 x 10^5) were nucleofected with 1 μg of plasmid encoding for the R02 CRISPR/Cas9 nuclease and then analyzed using a custom SMRT sequencing analysis pipeline. The change in the number of base pairs resulting from NHEJ repair of DNA cleavage in *HBB* was compiled for each sequence read. The y-axis represents the percentage of indels with specified number of base pair changes.

Gene	Sequence
HBB-F	AGGCACCGAGCACTTTCTTGCC
HBB-R	ACCCTGTGGAGCCACACCCTA
HBD-F	GAGGTTGTCCAGGTGAGCCAGGCCATCAC
HBD-R	CTGCTGAAAGAGATGCGGTGGGGAGATATGTA
GRIN3A-F	GTTTCTAAGAGCGGTGGCTCTCA
GRIN3A-R	CTGCCCCATCTATGCTTGGGA

Supplementary Table 1. Sequences of primers used to amplify the endogenous genes for the T7E1 mutation detection assays.

Gene	Sequence
GFP Integration-F	CGACAACCACTACCTGAGCA
GFP Integration-R	AGCAGAATGGTAGCTGGATTG
HBB Control-F	TGGTGGTGAGGCCCTGGGCAGGTTG
HBB Control-R	TAAAAGCAGAATGGTAGCTGGATT

Supplementary Table 2. Sequences of primers used for PCR confirmation of HDR-mediated GFP integration in *HBB*. The control primers for amplifying the *HBB* are also shown in the table.

Primer	Sequence
Beta 4F-Tag1	atcgAGGCACCGAGCACTTTCTTGCC
Beta 4F-Tag2	cagaAGGCACCGAGCACTTTCTTGCC
Beta 4F-Tag3	gctaAGGCACCGAGCACTTTCTTGCC
Beta 4F-Tag4	tgacAGGCACCGAGCACTTTCTTGCC
Beta 4F-Tag5	acgtAGGCACCGAGCACTTTCTTGCC
Beta 4F-Tag6	catgAGGCACCGAGCACTTTCTTGCC
Beta 4F-Tag7	gtgaAGGCACCGAGCACTTTCTTGCC
Beta 4F-Tag8	tagcAGGCACCGAGCACTTTCTTGCC
Beta 4F-Tag9	agtcAGGCACCGAGCACTTTCTTGCC
Beta 4R-Tag1	atcgACCCTGTGGAGCCACACCCTA
Beta 4R-Tag2	cagaACCCTGTGGAGCCACACCCTA
Beta 4R-Tag3	gctaACCCTGTGGAGCCACACCCTA
Beta 4R-Tag4	tgacACCCTGTGGAGCCACACCCTA
Beta 4R-Tag5	acgtACCCTGTGGAGCCACACCCTA
Beta 4R-Tag6	catgACCCTGTGGAGCCACACCCTA
Beta 4R-Tag7	gtgaACCCTGTGGAGCCACACCCTA
Beta 4R-Tag8	tagcACCCTGTGGAGCCACACCCTA
Beta 4R-Tag9	agtcACCCTGTGGAGCCACACCCTA

Supplementary Table 3. Sequences of primers used for amplifying *HBB* in single cell clones for the T7E1 assay and Sanger sequencing. Unique barcodes used to identify each clone is shown in lowercase.

	R02	L4-R4
Total Clones analyzed by T7E1	78	53
% Clones with on- and off-target activity	32.1	13.2
% Clones with on-target activity only	14.1	15.1
% Clones with off-target activity only	6.4	11.3

Supplementary Table 4. Analysis of clones with on- and off-target activity. Clones derived from single cells injected with R02 CRISPR/Cas9 or L4-R4 TALENs were analyzed for on- and off-

target activity using the T7E1 indel detection assay. The percentage of clones having on-target indels with and without off-target indels, or off-target indels only is shown in the table for each nuclease.
